# Advances in the Multi-Orthogonal Folding of Single Polymer Chains into Single-Chain Nanoparticles

**DOI:** 10.3390/polym13020293

**Published:** 2021-01-18

**Authors:** Agustín Blazquez-Martín, Ester Verde-Sesto, Angel J. Moreno, Arantxa Arbe, Juan Colmenero, José A. Pomposo

**Affiliations:** 1 Centro de Física de Materiales (CSIC, UPV/EHU) and Materials Physics Center MPC, Paseo Manuel de Lardizabal 5, E-20018 San Sebastián, Spain; agustinblazquezmartin@gmail.com (A.B.-M.); mariaester.verde@ehu.eus (E.V.-S.); angeljose.moreno@ehu.eus (A.J.M.); mariaaranzazu.arbe@ehu.eus (A.A.); juan.colmenero@ehu.eus (J.C.); 2 Donostia International Physics Center (DIPC), Paseo Manuel de Lardizabal 4, E-20018 San Sebastián, Spain; 3 Departamento de Polímeros y Materiales Avanzados: Física, Química y Tecnología, University of the Basque Country (UPV/EHU), PO Box 1072, E-20800 San Sebastián, Spain; 4 IKERBASQUE—Basque Foundation for Science, Plaza Euskadi 5, E-48009 Bilbao, Spain

**Keywords:** folding, interactions, single-chain nanoparticles

## Abstract

The folding of certain proteins (e.g., enzymes) into perfectly defined 3D conformations via multi-orthogonal interactions is critical to their function. Concerning synthetic polymers chains, the “folding” of individual polymer chains at high dilution via intra-chain interactions leads to so-called single-chain nanoparticles (SCNPs). This review article describes the advances carried out in recent years in the folding of single polymer chains into discrete SCNPs via multi-orthogonal interactions using different reactive chemical species where intra-chain bonding only occurs between groups of the same species. First, we summarize results from computer simulations of multi-orthogonally folded SCNPs. Next, we comprehensively review multi-orthogonally folded SCNPs synthesized via either non-covalent bonds or covalent interactions. Finally, we conclude by summarizing recent research about multi-orthogonally folded SCNPs prepared through both reversible (dynamic) and permanent bonds.

## 1. Introduction

**Figure 1:**
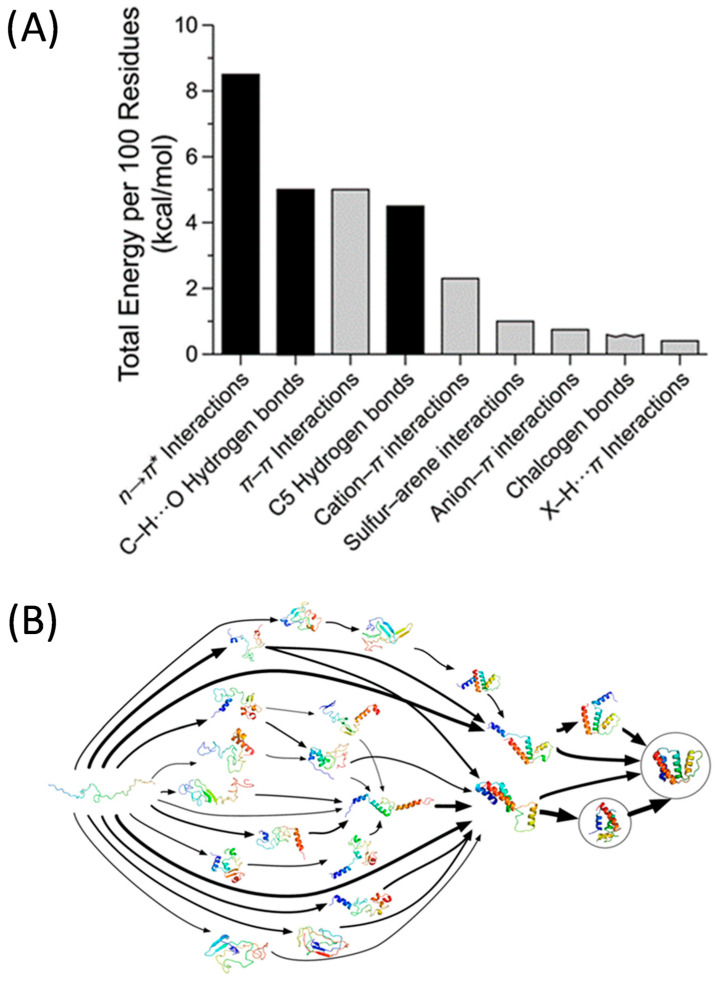
(**A**) Estimated enthalpic contributions of different interactions to the conformational stability of globular proteins: black bars, interactions of the main chain; gray bars, interactions involving side chains. Reprinted from reference [2] with permission. (**B**) Possible shapes and folding pathways that a protein can take as it condenses from its initial randomly coiled state (left) into its native 3D structure (right) as revealed from a Markov state model. Reprinted from Ref. [3] with permission.

The compaction process of a biological linear polypeptide chain to its functional, native conformation is called protein folding [1]. The folding of polypeptide chains is promoted by multiple non-covalent interactions. Canonical forces involved in protein folding are the hydrophobic effect, conventional hydrogen bonding, Coulombic interactions and van der Waals interactions. Moreover, secondary interactions of the polypeptide main chain (e.g., C–H⋯O hydrogen bonds, n→π* interactions, C5 hydrogen bonds) and side-chain atoms (e.g., cation-π interactions, π-π interactions) contribute largely to the conformational stability of a variety of folded proteins (Figure 1A). Because of the different, orthogonal interactions [2] involved, the free energy of the folded state of a typical globular protein is ca. 10–15 kcal/mol less than that of the unfolded state of the corresponding polypeptide chain (Figure 1B) [3].

Concerning synthetic polymers chains, the “folding” of individual polymer chains at high dilution via intra-chain interactions leads to so-called single-chain nanoparticles (SCNPs) [4,5,6,7,8,9,10,11,12,13,14,15,16,17,18,19]. In this case, the term “folding” refers to the process by which a functionalized synthetic polymer chain assumes its final shape or conformation as individual single-chain nanoparticle via non-covalent (or dynamic covalent) and/or covalent intra-chain interactions. In the case of SCNPs, folding, single-chain compaction and intra-molecular compaction are often utilized as synonyms. However, “unfolding” of SCNPs is formally restricted to SCNPs with non-covalent and dynamic covalent intra-chain interactions [16]. As summarized in Table 1, folding/unfolding of SCNPs has been investigated for a variety of reversible SCNPs with non-covalent bonds and dynamic covalent interactions [11], most of them involving a single interaction type (i.e., single-step crosslinking method). On one hand, the reversible supramolecular folding interactions investigated up to now include hydrogen bonding, host−guest complexation and hydrophobic-mediated self-assembly. On the other hand, a variety of dynamic (responsive) covalent bonds have been utilized, including hydrazone bonds, disulfide bridges, enamine bonds, etc. SCNP folding (and unfolding) was monitored mainly by dynamic light scattering (DLS), static light scattering (SLS), size exclusion chromatography (SEC), ultraviolet (UV) spectroscopy, fluorescence (FL), Fourier transform infrared (FTIR) spectroscopy, nuclear magnetic resonance (NMR) spectroscopy, circular dichroism (CD) and AFM-based single-molecule force spectroscopy.

Due to the concerted “multi-orthogonal” specific interactions (hydrophobic, hydrophilic, electrostatic, etc.) within the sequence of residues involved in protein folding, the compact native conformation of globular proteins is solid-like, and a scaling law *R* ≈ *N*^1/3^ is found where *R* is the protein size and *N* is the number of residues in the polypeptide chain [41]. Conversely, most of the SCNPs synthesized under good solvent conditions are far from being globular nano-objects even if prepared by using highly efficient “click” chemistry procedures, showing an approximate scaling law *R* ≈ *N^υ^* where *N* is the number of monomers in the polymer chain and the scaling exponent is υ~0.5 [42] (see discussion in Section 2). This scaling law resembles that observed for linear polymer chains in a theta-solvent or for intrinsically disordered proteins (IDPs) [43,44]. In spite of the rudimentary folding process observed in the case of synthetic polymer chains when compared to that found in proteins, the resulting morphology of SCNPs allows for appropriate immobilization of external molecules/metal ions/sensing probes. Remarkably, the entrapment process, which can be designed to be transient or permanent, opens exciting opportunities to develop efficient enzyme-mimic catalysts, new drug delivery nanosystems and fluorescent nanomaterials, among other applications [6].

Significant effort has been devoted in recent years to the development of protocols for the synthesis of SCNPs by means multi-orthogonal folding, in particular through the synergic combination of several experimental techniques and computer simulations with the aim of obtaining well-defined, unique SCNPs. Up to now, single chain folding in synthetic polymers does not generate the ‘perfect’ tertiary structure found in proteins. Only by combining almost monodisperse synthetic macromolecules with precise, controlled monomer sequences and multi-orthogonal interactions, a similar degree of perfection would be achieved. Earlier reviews on the topic of SCNPs focused on applications and characterization of these soft nanomaterials [5,6,7,8,9,10,11,12,13,14,15,16,17,18,19]. The current review aims to provide a new perspective on the synthesis of SCNPs by means multi-orthogonal folding. Future directions are outlined in the Conclusions.

## 2. Simulations of Multi-Folded Single-Chain Nanoparticles

The systematic use of computer simulations has provided a great advance in the current knowledge of the fundamental mechanisms controlling the molecular topology of SCNPs [6,45,46,47,48,49,50]. Coarse-grained models retaining the basic ingredients of the interactions (monomer excluded volume, non-crossability of the chain segments and backbone connectivity) can provide, at an affordable computational cost, a qualitative picture of the emerging broad physical scenarios. Molecular dynamics (MD) simulations of a simple bead-spring model of polymers [45] revealed that SCNPs prepared from monofunctional precursors in good solvent conditions (*R* ≈ *N*^0.63^) were not globular objects (*R* ≈ *N*^0.33^). The mean size *R* of the resulting SCNPs followed a power-law dependence *R* ≈ *N^υ^* on the number of monomers *N*, with an exponent υ~0.5. A compilation of experimental results in SCNPs with different chemical compositions and obtained through many different bonding protocols confirmed the simulation results [42]. The simulations revealed that even for the same precursor chain, the resulting SCNPs were topologically polydisperse, the distribution being dominated by sparse architectures with local globulation and at most a few long loops. There is a fundamental physical reason for this general observation [45]: the self-avoiding character of the linear precursor in the usual good solvent conditions for the synthesis promotes bonding between groups that are close in the linear backbone. Bonding events between groups separated by a long contour distance *s* are unfrequent (decaying in a similar way as in Gaussian chains [51], *P(s)*~*s*^−3/2^) and insufficient to fold the precursor into a globular SCNP. Increasing the number of reactive groups in the backbone has a minor effect on the size of the resulting SCNPs [45].

**Figure 2:**
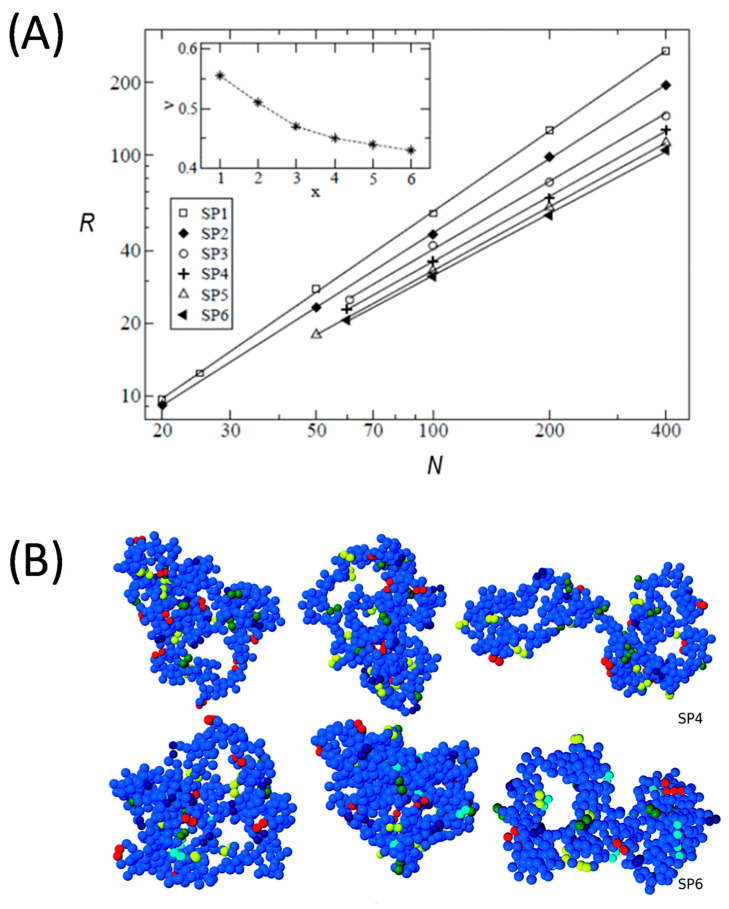
(**A**) Scaling behavior, as obtained from MD simulations according to *R* ≈ *N^υ^*, of SCNPs with different values of reactive chemical species *x* in which bonding is only possible between groups of the same species (denoted as SPx; *x* = 1, 2 and >2 correspond to monofunctional, orthogonal and multi-orthogonal protocols, respectively). The obtained scaling exponents *υ* are represented versus *x* in the inset. (**B**) Typical morphology of SCNPs obtained by multi-orthogonal folding: top line, SP4; bottom line, SP6. Dark blue beads correspond to inert monomers. Beads of other colors correspond to the reactive sites (a different color for each chemical species). Reprinted from Ref. [46] with permission.

The effect of introducing orthogonal chemistry on the conformations of the SCNPs was investigated through the former coarse-grained models for *x* = 2 different chemical species (A,B) in the reactive groups [45], and where only reactions within the same species (A-A or B-B) were allowed. The study was further extended to the multi-orthogonal case [46] by simulating synthesis for values up to *x* = 6. Figure 2A shows results [46] for the scaling behavior *R* ≈ *N^υ^* of SCNPs with the same number of monomers and reactive groups in the precursor but different numbers 1 ≤ *x* ≤ 6 of orthogonal species (data sets are accordingly denoted as SP*x*). The simulations for *x* = 2 compared the limit cases of simultaneous bonding (A-A and B-B reactions simultaneously allowed and with the same rate) and sequential bonding (B-B reactions occurring only after all A-A bonds were formed). Both limits only produced small differences in the size and shape of the SCNPs. As illustrated in Figure 2A more compact SCNPs were systematically obtained by increasing the number of multi-orthogonal species (see representative molecular architectures in Figure 2B), which led to a reduction of both the mean size and topological dispersity (this being quantified by the distribution of the asphericity and prolateness [46]). Shrinking at higher *x* was a direct consequence of increasing the mean distance between groups that could form mutual bonds (longer distance for higher *x*) which produced a higher fraction of long loops. However, this was still insufficient to obtain globular topologies (*υ* = 1/3), which were not approached even for the case *x* = 6 (for which having an experimental realization is currently far from feasible).

The simulations of References [45,46] explored the case of irreversible cross-links, i.e., every formed loop during the folding process was permanent. This condition creates steric interactions and entanglements that affect the subsequent intramolecular motions that take place during the completion of the cross-linking process. Monte Carlo (MC) simulations of the self-assembly of linear precursors into SCNPs using reversible intramolecular linkages, where loops can be formed and broken in a dynamic network, were carried out by Oyarzún and Mognetti [52]. Chains with different fraction *f* of reactive groups and association strength were investigated. The work was mainly focused on increasing the efficiency of sampling conformations of SCNPs and a quantitative characterization of their topological dispersity was not presented. The general conclusion for reversible strong association was that long loops were more frequent at high fraction of reactive groups, and a non-monotonic dependence of the SCNP size with *f* was found, the sparsest conformations being found at intermediate *f*. Though a reversible analogue of the irreversible systems of Reference [46] was not studied, the conclusions of Reference [52] are presumably expected for the case of reversible multi-orthogonal bonding.

In a different context but with consequences for the multi-orthogonal synthesis of SCNPs, simulations by Cardelli et al. [53] have addressed a fundamental question: Can we design artificial heteropolymers with chemical sequences that uniquely collapse into a stable target conformation, in a similar fashion to the case of globular proteins? According to mean field theories [54] the designability of a heteropolymer increases with the alphabet size (number of different chemical species in the chain) and decreases with the conformational entropy per monomer. The models of References [45,46] are good approximations for precursors with functionalized flexible side branches, which lead to effective isotropic interactions mediating the cross-linking, but such precursors do not fulfill the condition of low conformational entropy necessary for designability. Instead, this can be achieved by implementing directional interactions (e.g., H-bonding, π-π stacking, host-guest complexation) which can be introduced in simulations through “patchy” interactions. The conformational entropy is further reduced if backbone stiffness is introduced. Simulations of flexible and semiflexible heteropolymer models with an alphabet of *n* different species and *p* patchy interactions per monomer showed designability if some minimal values of *n* and *p* were imposed (*n* ≥ 3 and *p* ≥ 1 in the semiflexible model) [53]. This study strongly suggests that, irrespective of the number of chemical species in the chain, precursors without directional interactions cannot produce designable SCNPs (i.e., folded into a specific target structure) but a distribution of SCNPs polydisperse in size and shape. Unique SCNPs will presumably result by combining monodisperse synthetic macromolecules with precise, controlled monomer sequences and multi-orthogonal interactions.

## 3. Multi-Folded Single-Chain Nanoparticles via Non-Covalent Bonds

**Figure 3:**
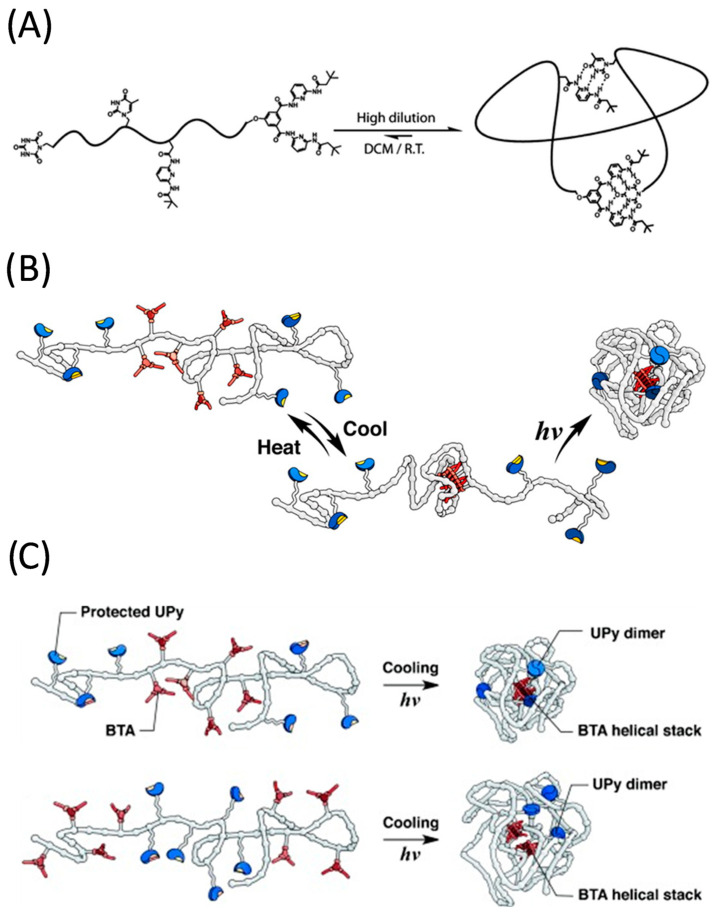
(**A**) Schematic illustration of the reversible single chain folding in dichlorometane (DCM) at room temperature (R.T.) and high dilution of a synthetic polymer chain containing two distinct and mutually orthogonal hydrogen bonding motifs (thymine–diaminopyridine and cyanuric acid–Hamilton wedge, respectively). (**B**) Sequential single chain folding of an ABA triblock copolymer at high dilution via a two-step thermal/photoirradiation treatment that resulted in the intramolecular formation of BTA-based helical aggregates (in red) and UPy dimers (in blue). (**C**) Illustration of the influence of block sequence (ABA vs. BAB) on the packing size of the resulting folded SCNPs via intramolecular formation of BTA-based helical aggregates (in red) and UPy dimers (in blue). Reprinted from Refs. [55,56,57] with permission.

With the long term aim of preparing macromolecules that mimic the folding actions of natural biomacromolecules, Barner-Kowollik and coworkers pioneered in 2012 the single chain folding of synthetic polymers containing two distinct and mutually orthogonal hydrogen bonding (HB) motifs: thymine (Thy)–diaminopyridine (DAP) and cyanuric acid (CA)–Hamilton wedge (HW) [55]. Starting from a CA-functionalized atom transfer radical polymerization (ATRP) initiator, a heterofunctional single polymer chain was prepared by ATRP followed by the insertion of a connector compound via modular ligation chemistry. After synthesis, each individual polymer chain was expected to contain (on average) only four complementary hydrogen bonding groups, corresponding to each of the different HB moieities. The resulting α-CA, Thy- and DAP-bearing, and ω-HW functionalized polymer chains were self-folded in dichloromethane (DCM) at low temperature and high dilution (Figure 3A) as revealed by dynamic light scattering (DLS) and ^1^H nuclear magnetic resonance (NMR) spectroscopy measurements. Static light scattering (SLS) results confirmed the single-chain nature of the self-folded macromolecules. A decreasing stability of the self-folded single polymer chains upon increasing temperature was observed through variable-temperature (VT) NMR experiments.

Independently, Palmans, Meijer and colleagues reported ABA triblock copolymers containing two complementary association motifs that fold into single-chain nanoparticles via orthogonal self-assembly [56]. The copolymers were composed of *o*-nitrobenzyl protected 2-ureidopyrimidinone (UPy) moieties in the A block, and benzene-1,3,5-tricarboxamide (BTA) units in the B block. To promote sequential single chain folding of the ABA triblock copolymers at high dilution, a two-step thermal/photoirradiation treatment was applied that resulted in the intramolecular formation of BTA-based helical aggregates and UPy dimers (Figure 3B). VT-NMR measurements in combination with circular dichroism (CD), size exclusion chromatography (SEC), small angle X-ray scattering (SAXS) and atomic force microscopy (AFM) experiments demonstrated the successful orthogonal (i.e., without mutual interference) self-assembly of BTA and UPy motifs, mimicking secondary-structuring elements in proteins. Subsequently, the folding process of ABA and BAB-type triblock copolymers comprising distinct BTA and UPy moieities in each block was investigated by this team [57]. Interestingly, an influence of block sequence on the folded structure was found, which affected the packing size of the resulting folded SCNPs. Hence, placing the UPy motifs in the middle block resulted in a more loose packing structure because BTAs self-assemble separately in both end blocks (Figure 3C).

Orthogonal hydrophobic and hydrogen bonding interactions were employed by Palmans, Meijer and coworkers for the construction of self-folding ruthenium-based catalytic systems in water [23]. Copolymers containing hydrophilic repeat units, diphenylphosphino-styrene units (as ligands of Ru ions) and complementary monomers leading to orthogonal hydrophobic self-assembly and intramolecular formation of BTA-based helical aggregates were synthesized. They were employed in the transfer hydrogenation of cyclohexanone in water with values of turnover frequency (TOF) of 10–20 h^−1^. A subsequent work, however, revealed that the presence of the BTA-based helical aggregates in these systems was not decisive for catalytic performance [58].

Photochemical isomerization and metal complexation of imine-containing polymers for intramolecular single-chain folding were introduced by the groups of Barner-Kowollik and Lehn with the aim to achieve orthogonally addressable/doubly stimuli-responsive systems [59].

**Figure 4:**
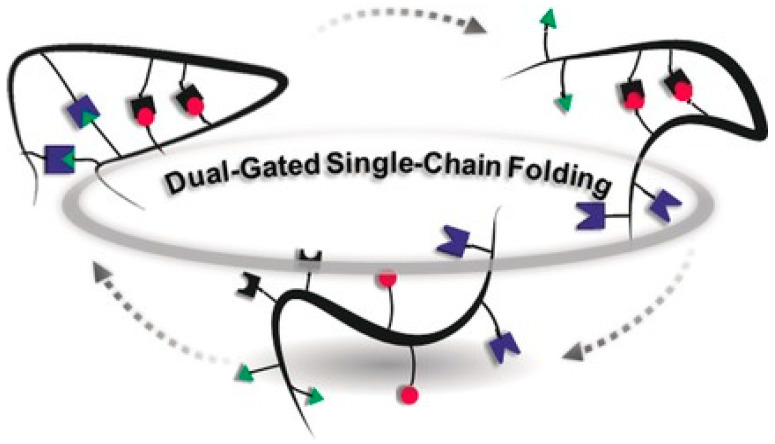
Illustration of a tetrablock copolymer equipped with mutually orthogonal folding elements (represented in different colors) allowing self-folding into a SCNP and orthogonal, order-independent unfolding of the SCNP in response to simple chemical triggers. Reprinted from Ref. [30] with permission.

The orthogonal, stepwise, and order-independent unfolding of SCNPs compacted by multiple hydrogen bonds and host-guest interactions was developed by Barner-Kowollik and coworkers based on tetrablock ABCD copolymers equipped with orthogonal recognition motifs [30]. Methanol was used to disrupt the hydrogen bonds, whereas KPF_6_ was employed to promote the decomplexation of the host-guest motif (Figure 4). The stepwise unfolding of the copolymer was evidenced by DLS and diffusion-ordered NMR spectroscopy (DOSY).

Self-folding polymers in both water and chloroform based on amphiphilic random copolymers containing 30–40 mol% of dual hydrophobic/hydrogen bonding urea pendants were described by Terashima, Sawamoto and coworkers [60]. Interestingly, only the random copolymers produced self-folded, compact SCNPs while gradient or block counterparts induced multi-chain aggregation. Moreover, these self-folded SCNPs showed on-demand folding/unfolding controllability by solvents, acidic/protic compounds and temperature. Hence, the folded structure of these SCNPs in water was dynamic and reversible: the mobility increased upon heating, and the structure unfolded by adding methanol but was efficiently reproducible even after suffering a lower critical solution temperature (LCST)-type phase separation process upon heating. In chloroform, these SCNP unfolded with addition of trifluoroacetic acid (TFA) due to an effective disruption of intramolecular hydrogen-bonding interactions by TFA. In a subsequent work by this team, amphiphilic random block copolymers with distinct hydrophobic pendants were self-assembled to provide SCNPs bearing double nanodomains. The compartmentalization was effectively achieved through phase separation of the hydrophobic pendants and intramolecular cross-linking within the self-folded block polymers in water [61].

**Figure 5:**
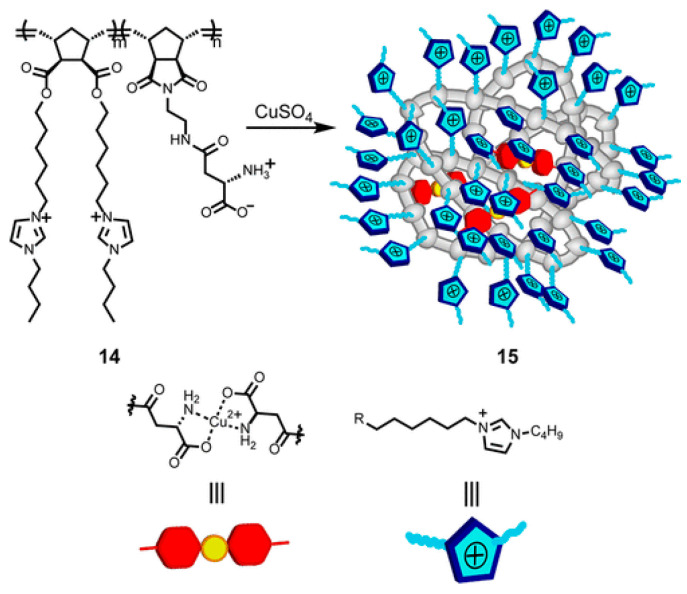
Combination of hydrophobic interactions, metal complexation and electrostatic charges for the construction of folded SCNPs for catalysis. Reprinted from Ref. [62] with permission.

The combination of hydrophobic interactions, metal complexation and electrostatic charges allowed Zimmerman and co-workers to develop a variety of dynamic SCNPs showing enzyme-like catalytic behavior (Figure 5) [62,63] and, even, able to perform tandem reactions together with enzymes inside living cells [64].

Independently, the presence of compartmentalized ultra-fine nanoclusters in metal folded SCNPs and subdomains in their assemblies was reported by Cai et al. [65], which used single chain technology to demonstrate unidirectional cross-domain molecule shutting of dumbbell-shaped SCNPs prepared by stepwise complexation of the outer blocks of an ABC-type linear triblock copolymer to copper ions [66].

Recently, random terpolymers decorated with two orthogonal ligand moieties, phosphines and phosphine oxides, were synthesized by Barner-Kowollik, Roesky and colleagues [67] for the formation of heterometallic Eu(III)/Pt(II) SCNPs entailing both catalytic and luminescent properties. The activity of the SCNPs as a homogeneous and luminescent catalytic system was demonstrated in the amination reaction of allyl alcohol.

**Figure 6:**
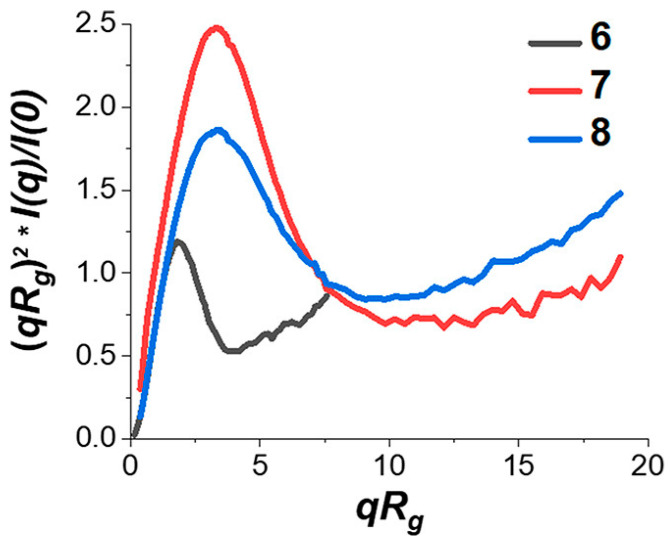
Comparison of the normalized SAXS Kratky plot of bovine serum albumin (BSA) (denoted as (6) with those of a PEG-functionalized random heteropolymer (7) and a PEG-functionalized block copolymer (8) both synthesized through high throughput PET-RAFT. Reprinted from Ref. [68] with permission.

High throughput photoinduced electron/energy transfer reversible addition−fragmentation chain-transfer (PET-RAFT) polymerization and high throughput SAXS were combined by Gormley and colleagues [68] to characterize the SCNP formation ability (compactness and flexibility) of a large combinatorial library (>450) of several homopolymers, random heteropolymers, block copolymers, PEG-conjugated polymers, and other polymer-functionalized polymers with varied composition and physicochemical characteristics (including the use neutral, hydrophilic, hydrophobic, and charged repeat units). Remarkably, only a small group (9/457) of PEG-functionalized random heteropolymers and block copolymers were found to exhibit compactness and flexibility similar to that of bovine serum albumin (BSA) by DLS, NMR, and SAXS (see Figure 6). In general, a rough association between compactness and flexibility parameters (*R*_g_/*R*_h_ and Porod exponent, respectively) with log *P*, a quantity that describes hydrophobicity, helped to demonstrate and predict material parameters leading to SCNPs with greater compactness, rigidity, and stability [68].

In the near future, it is expected that advances in simulation techniques combined with high throughput synthetic methodologies and precise characterization techniques at chain and sub-chain levels will allow to cover the large parameter space available to produce SCNPs folded with “at will” compactness and rigidity via multi-orthogonal non-covalent interactions.

## 4. Multi-Folded Single-Chain Nanoparticles via Covalent Bonds

The orthogonal folding of sequence-controlled macromolecules into compartmentalized SCNPs containing distinct cross-linked subdomains was reported in 2014 by Roy and Lutz [69]. The folded SCNPs were obtained by stepwise, orthogonal intramolecular cross-linking of appropriate sequence-controlled precursors containing two individually addressable cross-linking zones separated by an inert (non-reactive) polymer region. Cross-linking of the first zone of the precursor was carried out through reaction of pentafluorophenyl activated ester moieties of the precursor with ethylenediamine as external cross-linker. The second zone of the precursor was cross-linked through intra-chain alkyne homocoupling after deprotection of the alkyne functionalities of the precursor. The stepwise compaction process was investigated by NMR spectroscopy and SEC.

Subsequently, Perrier and co-workers developed a method to produce sequence-controlled multiblock SCNPs based on a simple, stepwise folding-chain extension-folding process [70]. The approach was used to synthesize a complex pentablock copolymer having three individually folded subdomains with an overall dispersity of 1.21. The formation of SCNPs was confirmed by SEC, NMR, differential scanning calorimetry (DSC) and AFM.

The use of two competitive photochemical reactions to induce the intra-chain cross-linking of linear polymer chains into SCNPs was reported by Barner-Kowollik and coworkers [71]. Precursor polymers containing tetrazole, alkene and acrylic acid functional groups were found to fold via dual nitrile imine-mediated tetrazole-ene cycloaddition (NITEC) and nitrile imine-carboxylic acid ligation (NICAL). In a subsequent work by this team, a versatile visible light driven process for the preparation of fluorescent SCNPs via tetrazole-based mechanisms (NITEC, NICAL, as well as self-dimerization) was introduced [72].

Stepwise light-induced dual compaction of SCNPs was investigated by the groups of Perrier and Barner-Kowollik [73] using ABC triblock copolymer precursors decorated with phenacyl sulfide (A-block) and photoenol (C-block) moieties. UV irradiations at 355 and 320 nm were performed to induce the cross-linking of the phenacyl sulfide and photoenol domains in the presence of dithiol and diacrylate external linkers, respectively. Asymmetrical compaction steps were observed for these triblock copolymers, with the first folding step significantly exceeding the second one in magnitude. This behavior was tentatively attributed to the significant loss of conformational degrees of freedom accompanying the first folding step.

Amphiphilic Janus twin SCNPs were synthesized by Zhao and co-workers [74] based on amphiphilic AB diblock copolymers containing antracene (A-block) and bromine (B-block) groups, respectively. Janus twin SCNPs were obtained after two-step independent intramolecular cross-linking reactions based on anthracene photodimerization and an atom transfer radical coupling (ATRC) reaction, respectively. The resulting SCNPs were able to reduce the surface tension of water due to their self-assembly in aqueous solution into vesicles with the hydrophobic particles in the inner walls and the hydrophilic particles on the surfaces.

**Figure 7:**
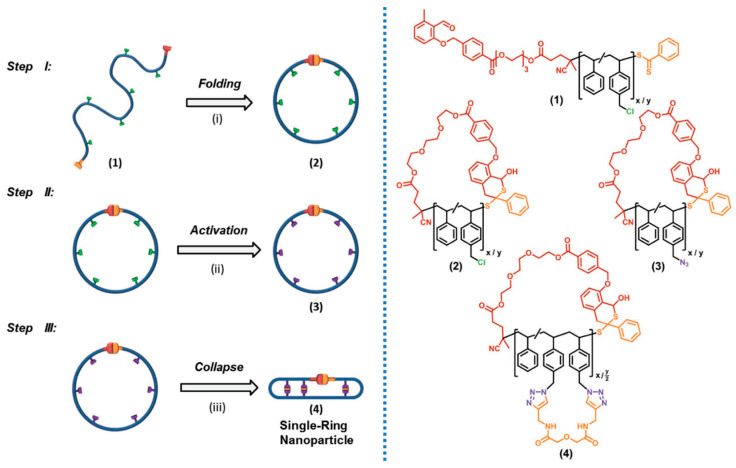
Schematic illustration of the synthesis of single-ring nanoparticles (SRNPs) mimicking natural cyclotides by a stepwise folding (I)-activation (II)-collapse (III) process, and chemical structure of the precursor polymer chains (1), intermediates (2, 3) and SRNPs (4). Reprinted from Ref. [75] with permission.

The synthesis of single-ring nanoparticles (SRNPs) mimicking natural cyclotides by a stepwise folding-activation-collapse process at high dilution starting from simple synthetic precursor polymers was reported by Barner-Kowollik, Pomposo and co-workers (Figure 7) [75]. The initial folding step was carried out by a photoactivated hetero Diels- Alder (HDA) ring-closing reaction, which was accompanied by chain compaction of the individual precursor polymer chains as determined by SEC. The subsequent activation step comprised a simple azidation procedure, whereas the final collapse step was driven by classical “click” chemistry (CuAAC) in the presence of an external dialkyne cross-linker, providing additional compaction to the final SRNPs. The unique structure and compaction degree of the SRNPs was established via a detailed comparison with conventional SCNPs prepared exclusively by chain collapse from the exact same precursor polymer (without the pre-folding step). Hence, the unique structure and compaction degree of the SRNPs as cyclotide mimetics was revealed by their significantly higher shrinking factor, G = 0.61, when compared to that of conventional SCNPs, G = 0.87, synthesized from exactly the same precursor polymer but without involving the first folding step.

A dual photoreactive precursor polymer entailing equal number of antracene and styrylpyrene units was synthesized by Barner-Kowollik and colleagues [76]. This polymer enabled facile access to two distinct states of single chain folding depending exclusively on the color of visible light used to promote the [4+4] photocycloadditon of anthracene and the [2+2] photocycloadditon of styrylpyrene. Hence, upon irradiation with blue light the styrylpyrene units were selectively dimerized achieving the first state of single chain folding. The second one was accessed by switching the irradiation wavelength to violet light to induce the dimerization of the unreacted anthracene units. Conversely, direct irradiation of the photoreactive precursor polymer with violet light was found to induce both photoreactions at the same time. However, at early stages the folding was dominated by the styrylpyrene photodimerization due to the higher reactivity of the styrylpyrene group under such irradiation conditions.

**Figure 8:**
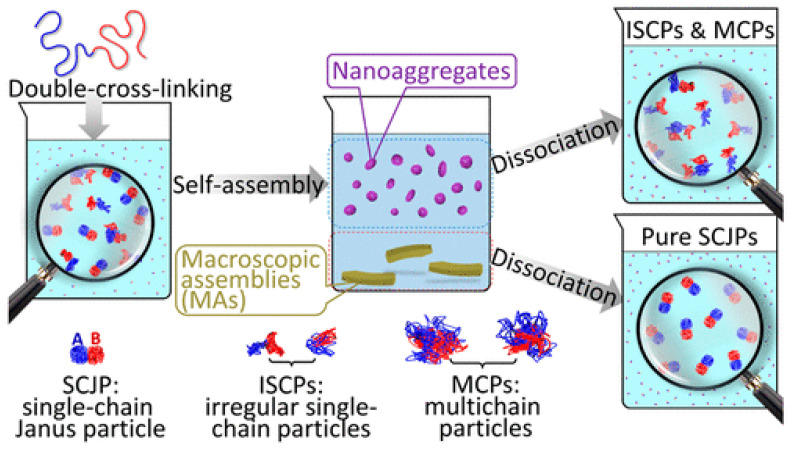
Facile isolation of pure single-chain Janus particles (SCJPs) by sorting through formation of macroscopic assemblies (MAS) via exclusive self-assembly (ESA). Reprinted from Ref. [77] with permission.

Dimers of SCNPs of high purity were isolated by sorting via exclusive self-assembly (ESA) by Chen and coworkers (Figure 8) [77]. First, single-chain Janus particles (SCJPs) were prepared by double cross-linking a diblock copolymer in methanol as the common solvent. The first block was cross-linked via Glaser coupling of the pendent alkyne groups, whereas the second one was cross-linked through a quaternization reaction. Inevitably, the double cross-linking led to a mixture containing not only SCJPs but also multi-chain particles and irregular single-chain particles. Under well-controlled conditions, the SCJPs in the mixture were found to self-assemble with high exclusivity to form regularly structured macroscopic assemblies (MAs) with a crystal-like appearance that precipitate from the suspension. Hence, pure SCJPs that were uniform in size, shape and Janus structure were efficiently prepared by collection and dissociation of these MAs.

**Figure 9:**
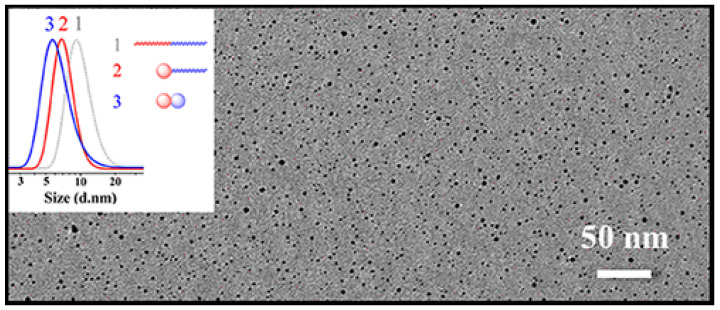
Dimers of SCNPs prepared in highly concentrated solutions through electrostatically-mediated intramolecular cross-linking of diblock copolymers as observed by TEM. The inset shows the progressive reduction in average hydrodynamic radius upon the sequential folding of each block as determined by DLS. Reprinted from Ref. [78] with permission.

Independently, dimers of SCNPs were prepared in highly concentrated solutions (up to 100 mg/mL) by Yang and coworkers through electrostatically-mediated intramolecular cross-linking of diblock copolymers [78]. The synthesis was orthogonally performed in a highly polar solvent such as DMSO. A poly(isoprene)-*b*-poly(vinylpyridine) (PI-*b*-P4VP) diblock copolymer was selected containing two different cross-linkable blocks for sequential cross-linking. In the first step, the PI block was modified with 2-mercaptoethylamine hydrochloride to introduce an electrostatic interaction along the chain. The intramolecular cross-linking was performed with 1,6-hexanediisothiocyanate. In the second step, the P4VP block was reacted with iodoethane to introduce an electrostatic interaction along the P4VP chain. Afterward, the intramolecular cross-linking was performed with 1,5-diiodopentane. A dimer of SCNPs was thus achieved whose two domains were different in composition: the PI domain containing residual amine and cyanate groups, and the P4VP domain containing residual pyridine and quaternized groups (Figure 9).

Attempts to produce highly compact SCNPs folded via two orthogonal procedures were recently carried out by Temel and coworkers [79]. A first folding step was carried out via click chemistry [80] involving an azide-decorated polymer precursor and dipropargylated benzophenone as external linker. After intra-chain cross-linking, the resulting SCNPs showed the characteristic UV absorption band of the benzophenone junction points. The second folding step was performed via UV irradiation to promote the transformation of the benzophenone moieties of the SCNPs into highly reactive ketyl radicals and subsequently to produce radical-radical coupling events. Although an increased compaction degree was observed via SEC measurements, both DSL and TEM results revealed the additional presence of a significant amount of multi-SCNPs aggregates.

Similar to the case of multi-folded SCNPs via non-covalent bonds, attempts to produce more compact SCNPs through the involvement of multiple covalent bonds are expected to grow in next years. One significant advantage of the use of covalent bonds is that robust SCNPs for a variety of applications are obtained [4]. However, it should be bear in mind that the internal dynamics of covalent-bonded SCNPs is expected to be slower than that of SCNPs with non-covalent bonds.

## 5. Multi-Folded Single-Chain Nanoparticles via Covalent and Non-Covalent Bonds

Multi-folded SCNPs via covalent and non-covalent interactions offer the advantage of being partially dynamic and responsive due to the presence of non-covalent bonds but retaining most of the stability imparted by the covalent interactions.

Berda and coworkers pioneered the use of multiple sequential orthogonal intra-chain covalent and non-covalent interactions for the controlled folding of a novel electroactive polyolefin [81]. A poly(oxanorbornene anhydride-*co*-cyclooctadiene) copolymer synthesized via ring-opening metathesis polymerization (ROMP) was used as SCNP precursor polymer. In a first step, the anhydride handles of the copolymer were decorated with an aniline tetramer (AT) that induced folding via supramolecular interactions between the AT pendant groups. The remaining anhydride groups were cross-linked with *p*-aminoaniline as external linker providing the second folding step. The last folding step was carried out in the presence of a dithiol linker via thiol-ene click chemistry involving the cyclooctadiene repeat units of the copolymer. Overall, the hydrodynamic volume of the SCNP was 70% smaller than that of the original coil.

**Figure 10:**
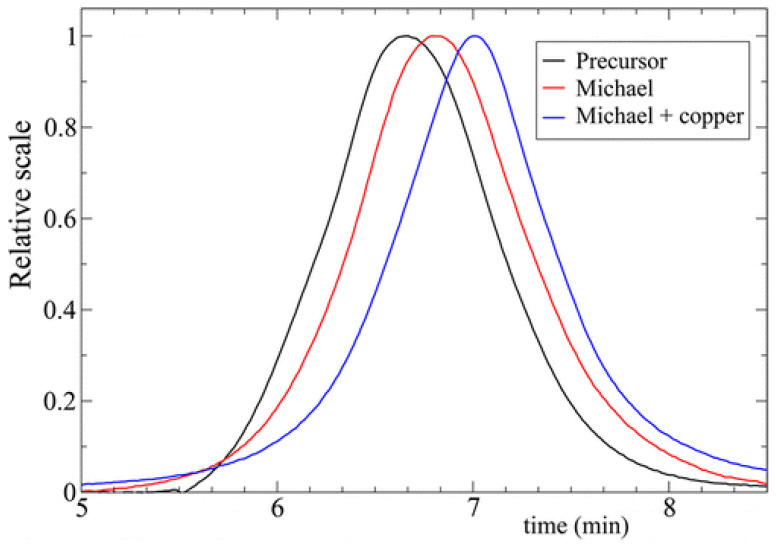
SEC traces of the precursor functionalized polymer (black), SCNPs synthesized from this precursor exclusively via covalent interactions (red) and SCNPs synthesized from this precursor via covalent and non-covalent interactions (blue). Reprinted from Ref. [45] with permission.

The advantages of orthogonal folding of single polymer chains to soft SCNPs via covalent and non-covalent interactions was further highlighted in real polymeric nanoparticles folded via sequential intra-chain Michael addition reaction and copper complexation [45]. Precursor polymers containing *β*-ketoester units were synthesized via reversible addition-fragmentation chain transfer (RAFT) polymerization. The experimental results obtained from SEC measurements (Figure 10) and SAXS experiments were supported by results of computer simulations (Table 2). Interestingly, MD simulations revealed that simultaneous and sequential cross-linking lead to the same structural properties of the resulting SCNPs. Additionally, SCNPs synthesized via orthogonal folding were found to show on average more globular conformations.

A useful strategy to improve the compaction of SCNPs is to combine covalent cross-linking with secondary non-covalent interactions to promote nanoparticle folding more akin to natural materials. This approach was first implemented in SCNPs in which external linkers were used to promote covalent cross-linking and the new cross-linking points were endowed with mutual hydrogen bonding interactions (e.g., bisurea moieties [82], amide groups [83]). Recently, this strategy has been further refined by Berda and colleagues by generating *α*-hydrazido dipeptides within hydrazone-cross-linked SCNPs [84]. More recently, Simon and coworkers have exploited hydrophobic interactions in comb copolymers to increase its compaction to SCNPs via intra-chain photodimerization [85].

## 6. Conclusions and Outlook

Given the initial success obtained when utilizing orthogonal and multi-orthogonal approaches for the synthesis of SCNPs, a variety of SCNPs folded via combination of multiple covalent and non-covalent interactions can be envisioned for next years. The basic understanding of how to integrate such a diversity of interactions into valuable polymeric precursors to produce SCNPs of different topologies is being generated, and it should aid and expedite progress in this evolving field. Realistic coarse-grained simulations will contribute decisively to facilitate and simplify the appropriate experimental protocols. An area to develop in next years is the use of machine-learning (ML) techniques for the computational design of SCNPs, i.e., (i) generating data of a given property at a moderate computational cost through the simulation in a reduced space of chemical sequences, (ii) using this information to train the ML model, and (iii) predicting and optimizing the property out of the space generated in step (i). Recent work has applied this strategy, which should be transferrable to SCNPs, to target specific molecular conformations of heteropolymer models (globular, swollen, rod-like, etc.) using the size distribution of a small number of simulated sequences [86]. As aforementioned, designability (folding into a unique stable state) might be approached by using bonding methods with directional interactions [53]. Additionally, significant advantages might arise from synergies with the emerging field of sequence-controlled polymers. Globular and multi-compartmentalized SCNPs—as a result of modern single chain technology—will be undoubtedly building blocks for a broad range of applications, including enzyme-mimetic catalysis, sensing, and drug delivery nanosystems, among other ones.

## Figures and Tables

**Table 1:** Reversible “folding”/“unfolding” of SCNPs.

Type ^1^	Folding Interaction ^2^	Characterization Technique ^3^	References
NCBs	Multiple hydrogen bonding	DLS	[20]
NCBs	UPy dimerization	SEC, AFM	[21,22]
NCBs	BTA helical stacking	UV, CD	[23]
NCBs	CB[8]-viologen complexation	FL	[24]
NCBs	Hydrophobic interactions	DLS, FL	[25,26]
NCBs	BTA helical stacking & CA−HW dimerization	NMR, CD, DLS, SLS	[27]
NCBs	*β*-CD-FC complexation	DLS, NMR	[28,29]
NCBs	B21C7-AS dimerization & CA−HW dimerization	DLS, NMR	[30]
NCBs	C4P-TD complexation	FL	[31]
NCBs	*β*-CD-AD complexation	NMR, DLS	[32]
DCBs	Hydrazone bonds	SEC	[33]
DCBs	Disulfide bonds	SEC	[34,35]
DCBs	Enamine bonds	SEC, FTIR	[36]
DCBs	Amine quaternization	SEC, DLS	[37]
DCBs	Boronate ester formation	SEC, DLS	[38]
DCBs	HDA reaction	SEC, DLS, NMR	[39]
DCBs	Photoligation of nitroxide radicals	SEC, DLS, NMR, FL	[40]

^1^ NCBs = Noncovalent bonds. DCBs = Dynamic covalent bonds. ^2^ UPy = 2-Ureido-pyrimidinone. BTA = benzene-1,3,5-tricarboxamide. CB[8] = Cucurbit[8]uril. CA = Cyanuric acid. HW = Hamilton wedge. *β*-CD = *β*-Cyclodextrin. FC = Ferrocene. B21C7 = Benzo-21-crown-7. AS = Ammonium salt. C4P = Calix[4]pyrrole. TD = Terephthalate dianion. AD = Adamantane. HDA = Hetero Diels-Alder. ^3^ DLS = Dynamic light scattering. SEC = Size exclusion chromatography. AFM = Atomic force microscopy. UV = Ultraviolet spectroscopy. CD = Circular dichroism. FL = Fluorescence. NMR = Nuclear magnetic resonance spectroscopy. SLS = Static light scattering. FTIR = Fourier transform infrared spectroscopy.

**Table 2:** Scaling exponent for the precursor, SCNPs synthesized via covalent interactions and SCNPs synthesized via covalent and non-covalent interactions as determined from MD simulations and SAXS measurements, according to *R* *≈ N^υ^* (*R* = size, *N* = polymerization degree, *υ* = scaling exponent) [45].

System	*υ* (Simulations)	*υ* (SAXS)	*R*(SEC), nm
Precursor	0.63	0.60	25.7
SCNPs (only covalent interactions)	0.56	0.44	19.6
SCNPs (covalent & non-covalent interactions)	0.51	0.40	10.6
